# Management of canine leishmaniosis in endemic SW European regions: a questionnaire-based multinational survey

**DOI:** 10.1186/1756-3305-7-110

**Published:** 2014-03-24

**Authors:** Patrick Bourdeau, Manolis N Saridomichelakis, Ana Oliveira, Gaetano Oliva, Tina Kotnik, Rosa Gálvez, Valentina Foglia Manzillo, Alex F Koutinas, Isabel Pereira da Fonseca, Guadalupe Miró

**Affiliations:** 1 Ecole Vétérinaire de Nantes, Oniris, Université de Nantes, Atlanpole, La Chantrerie, BP 40706 Nantes, France; 2 Clinic of Medicine, Faculty of Veterinary Medicine, University of Thessaly, Karditsa 43100, Greece; 3 Faculdade de Medicina Veterinária, Universidade Lusofona de Humanidades e Tecnologias, Campo Grande, 1729-024 Lisboa, Portugal; 4 School of Veterinary Clinical Sciences, University Federico II, Via Federico Delpino 1, 80137 Naples, Italy; 5 Small Animal Clinic, Veterinary Faculty of Ljubljana, C. v Mestni log 47, 1000 Ljubljana, Slovenia; 6 Departamento de Sanidad Animal, Facultad de Veterinaria, Universidad Complutense de Madrid, Avda. Puerta de Hierro s/n, 28040 Madrid, Spain; 7 Companion Animal Clinic, Faculty of Veterinary Medicine, Aristotle University of Thessaloniki, Thessaloniki 54627, Greece; 8 Faculdade de Medicina Veterinária, Pólo Universitário Alto da Ajuda, Universidade de Lisboa, Lisboa 1300-477, Portugal

**Keywords:** Canine leishmaniosis, Survey, Clinical signs, Diagnosis, Therapy, Prevention, Europe

## Abstract

**Background:**

Canine leishmaniosis (CanL) caused by *Leishmania infantum* is a widespread endemic disease in SW Europe. This study was designed to determine how veterinarians clinically manage CanL in this region by analysing information collected in a questionnaire completed by local veterinarians working in clinics in France, Portugal, Greece, Spain, Italy and Slovenia.

**Methods:**

Over the period 2004–2011, a questionnaire on CanL was sent to 12,546 small animal clinics located in the six countries surveyed. The questionnaire with 10 items comprising open and closed questions sought to obtain comparable data regarding the main clinical manifestations of CanL, the diagnostic methods used, the treatment regimens selected, recommended preventive measures and awareness of the important public health implications of CanL.

**Results:**

The data collected reflect similarities in the clinical manifestations reported although there was some variation in the concurrent diseases described, and wide variation in the clinical management of CanL among the countries examined in terms of dosing regimens, therapeutic agents and the criteria used to diagnose CanL. Most veterinarians properly informed dog owners about the preventive measures available and about the zoonotic implications of CanL.

**Conclusions:**

This survey describes the current situation in SW endemic countries in Europe regarding the clinical management of CanL. The data collected reveal a need to unify criteria from evidence-based medicine to determine and similarly apply the best diagnostic and treatment methods available for this disease in the different countries.

## Background

Leishmaniosis is a disease caused by several species of protozoan parasites of the genus *Leishmania* Ross, 1903 [1,2]. It is transmitted to humans and animals by blood-feeding female phlebotomine sand flies [3,4]. *Leishmania infantum* causes a severe zoonotic disease in dogs and humans [5], the domestic dog being considered the main reservoir for human infection by this species [6,7]. Due to the zoonotic nature of the disease, dogs infected with *L. infantum* are a serious concern for both animal and public health [8]. The high incidence of canine leishmaniosis (CanL) in SW Europe is well known [5] and it has been estimated that at least 2.5 million dogs could be infected (16.7%) [9]. The disease also seems to be spreading from the Mediterranean basin into northern Europe [10,11]. This northward spread has been attributed to climate change and globalisation (mainly the movement of dogs between countries) in the past decade [12]. These factors could have led to new disease foci in the north of classic endemic regions throughout Europe such as the foothills of the Alps [13] and the Pyrenees [14]. Moreover, there have been recent outbreaks of human leishmaniosis in classic endemic regions like Madrid, Spain [15,16].

In endemic areas, the number of infected dogs is considerably higher than the rate of apparent clinical disease [17]. Clinical CanL is a chronic systemic disease characterized by a variable and nonspecific spectrum of signs of different severity determined by the host immune response [8,18]. To describe the wide variety of clinical manifestations of CanL and provide a tool to determine the best treatment options and prognosis, a classification scheme defining four clinical stages based on clinical signs, clinicopathological abnormalities and serological status has been proposed by the LeishVet group [19]. According to this scheme, the main clinical signs of CanL are one or more of the following: weight loss, lethargy, muscular atrophy, non-regenerative anaemia, generalized lymphadenomegaly, splenomegaly, renal disorders, ocular lesions, arthropathies, onycogryphosis and skin lesions [8,19]. This broad spectrum of clinical signs means that the list for a differential diagnosis of CanL is extensive [19,20] and an integrated approach is needed to correctly diagnose the disease. This approach must be based on the following information: epidemiological data, clinical examination, complete blood count (CBC), biochemical profile, urinalysis, quantitative serological techniques and microscopy observation of *Leishmania* amastigotes in target tissues (lymph nodes, skin, and/or bone marrow) [21]. Currently available antileishmania drugs improve clinical signs after treatment, but parasitological cure is not attained [22-25]. The clinical response to treatment can vary depending on the initial clinical status and the specific response to therapy of each animal [19]. Combinations of meglumine antimoniate or miltefosine with allopurinol (a leishmaniostatic agent) have been widely used and considered first line treatment against CanL since they are the most effective [19,26-28]. Other drugs have been also used such as amphotericin B, aminosidine, pentamidine, metronidazole and spiramycin combined, enrofloxacin, marbofloxacin, ketoconazole and oleylphosphocholine with variable efficacy [18,23,29]. More recently, the immunomodulator domperidone has been approved for veterinary use and could be useful to treat non-severe clinical cases [30].

According to the results of both laboratory and field studies [31-35], the best way to reduce the spread of *L. infantum* infection in endemic areas is to protect dogs with topical insecticides [18,36]. Topical formulations against sand flies on dogs are usually spot-on or collar form products. The efficacy of several repellents applied to dogs has been recently reviewed [36].

In addition, several vaccine candidates against CanL infection have been recently tested [36-38]. In Europe, a vaccine based on the secreted-excreted antigen of *L. infantum* (CaniLeish, Virbac Animal Health) has been recently licensed [39], and has been available in some European countries since 2011. It is claimed that the CaniLeish vaccine induces cell-mediated immunity, promoting a Th1 response [40,41]. However, large-scale field studies are needed to determine if this vaccine will help control CanL in Europe.

To date, our understanding of CanL infection in Europe has been limited by a lack of comparable field data. Moreover, many clinical studies have used different approaches, sometimes very specialized, that do not really reflect the field situation.

This paper describes a survey conducted to assess the current situation of CanL in SW Europe based on a standardized questionnaire administered to local veterinarians with clinics in six countries: France, Portugal, Greece, Spain, Italy and Slovenia. Our survey provides important information concerning the clinical aspects and management of CanL in this region of Europe.

## Methods

A questionnaire to obtain information on CanL was initially created and tested in France, and then validated by academics from renowned European Veterinary Schools. The items included were based on the experience and results of a previous study [42]. The questionnaire was translated to each of the languages of the countries included in the study and sent to 12,546 small animal clinics successively in the six countries over the period 2004–2011. To avoid duplication of data, one questionnaire was sent per practice.

The questionnaire was designed to obtain the following data: the number of dogs examined per year at the clinic, the number of cases of CanL diagnosed in the past year, the frequency and main clinical signs of CanL detected, the frequency of concurrent diseases, the diagnostic method of choice, the treatment regimen used, survival times of animals after treatment, preventive measures recommended and the veterinarian’s opinion about the use of effective vaccines and public health implications.

## Results

### Reply rate, veterinary clinic clientele and frequency of CanL cases

Of 12,546 questionnaires initially sent, 2099 were completed and returned to give an overall reply rate of 16.7%. Reply rates for the six countries in descending order were: Slovenia 46.7% (49/105), France 17.9% (994/5567), Portugal 17.8% (141/790), Greece 17.6% (201/1144), Spain 16.1% (483/3000) and Italy 11.3% (231/2040). In France, 30.1% of replies were issued from the endemic part of the country and 69.9% from the non endemic area (eg, the participation of veterinary clinics in France from endemic areas was 14.3 to 42.9% and in the non endemic areas from 3 to 33%).

The first part of the questionnaire was designed to characterize the clientele of the different practices. The first item was: “Number of dogs per year admitted at least once”. There were four response categories for this question: <1000 (under 19 dogs per week); 1000–2500 (19 to 48 dogs per week); 2500–5000 (48 to 96 dogs per week) and >5000 dogs (more than 96 dogs per week) (Table 1). France and Spain have mainly medium and medium-large sized clinics (69.4% and 61.7% respectively). Large veterinary hospitals admitting more than 5000 dogs per year are only found in France (8.5%), Spain (2.1%) and Portugal (2%). Italy, Greece, Portugal and Slovenia have mostly small and medium sized clinics (93.8%, 92.9%, 92% and 79.6% respectively).

**Table 1:** Number of dogs admitted at least once per year to the veterinary clinics

**Number of clients (%)**	**France**	**Greece**	**Italy**	**Slovenia**	**Spain**	**Portugal**
**<1000**	22.1	64.8	62.1	59.2	36.1	65
**1000-2500**	40.1	28.1	31.7	20.4	35.1	27
**2500-5000**	29.3	7.1	6.1	10.2	26.6	6
**>5000**	8.5	0	0	0	2.1	2

We asked the clinicians to provide an average number of CanL cases per year calculating the mean of the last two years. The total number of CanL cases includes newly diagnosed cases. As indicated in Table 2, three countries in endemic areas for CanL reported a higher incidence of CanL: Greece, Spain and Portugal. Although Italy is also a CanL endemic country, unfortunately the information on this issue was missing. France has both endemic and non-endemic areas. The CanL cases in France were thus divided into those reported by veterinarians whose practices were in endemic and non-endemic areas. For Slovenia, very few positive CanL cases, mostly imported, were reported.

**Table 2:** Mean number of dogs with CanL observed on a yearly basis in the veterinary clinics

**CanL cases (%)**	**France a/b**	**Greece**	**Italy**	**Slovenia**	**Spain**	**Portugal**
**0**	46.1 / 8.7	1.5	nd	73.5	5.1	7.1
**1-5**	29.8 /32.1	15.1	nd	12.2	24.9	27
**5-10**	7.4 / 18	15.6	nd	0	14.7	10
**10-20**	6.9 / 17	22.6	nd	2*	21.3	19
**20-50**	5.6 / 13.9	30.7	nd	0	21.1	21
**>50**	4.2 / 10.3	14.5	nd	0	12.7	16

(a) Veterinarians in general; (b) Veterinarians working in endemic areas; (*) Breeder with 50 Basset Hounds originally from Italy; **nd**: not determined.

### Frequency of clinical signs of CanL and concurrent diseases

**Figure 1:**
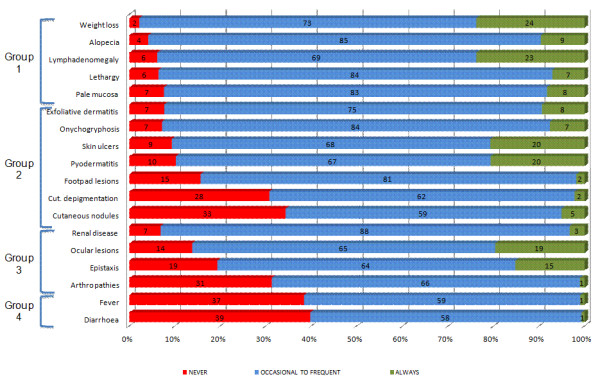
Frequencies of the clinical signs of CanL reported by veterinarians from Italy, Portugal, Spain, Greece and France.

Table 3 provides the frequencies of clinical signs of CanL on which the veterinarians based their suspicion of disease in each country. Clinical signs were categorized as: 1-early detectable signs; 2- skin lesions; 3- signs related to immune complex deposition; or 4- unusual signs (Figure 1). When we looked at the factors mainly considered for CanL diagnosis, weight loss, alopecia, lymphadenomegaly, lethargy, pale mucosa and some skin lesions emerged as those most frequently observed, whereas diarrhoea and fever were less frequently reported. 37% and 39% of veterinarians never observed fever and diarrhoea, respectively. Renal disease was widely described and is thought to be of great diagnostic value. Ocular lesions, epistaxis and arthropathies were less frequently observed but their presence is of high unfavourable prognostic value.

**Table 3:** Percentages of clinical signs on which veterinarians based their suspicion of CanL

	**France n = 994**			**Greece n = 201**			**Italy n = 231**		
**Clinical signs %**	**Never**	**Occasional to frequent**	**Always**	**Never**	**Occasional to frequent**	**Always**	**Never**	**Occasional to frequent**	**Always**
**Weight loss**	4.8	74.8	20.4	0.5	73.7	25.8	0.9	80.4	18.7
**Alopecia**	12.3	78.3	9.4	2.8	92.7	4.5	2.3	86.7	11
**Lymphadenomegaly**	9.1	70.6	20.3	0.5	70.8	28.6	4.6	71.2	24.2
**Lethargy**	13.2	78	8.8	4	88.4	7.5	5.1	87	7.9
**Pale mucosa**	21.3	73.2	9.4	1.1	87	12	4.2	88.8	7
**Exfoliative dermatitis**	21.6	35.8	6.8	4.8	91.1	4.1	1.9	87.6	10.5
**Onychogryphosis**	14.1	77.8	8	5	90	5	6.8	83.1	10
**Skin ulcers**	22.7	72.9	4.4	5.5	89	5.5	4.7	12.3	83
**Pyodermatitis**	21	73.3	5.7	12.5	86.9	0.6	0.5	11.7	87.8
**Footpad lesions**	20.1	76.9	3	19.6	80.4	0	12.7	85.4	1.9
**Cutaneous depigmentation**	49.8	48.7	1.5	34	66	0	33	64.6	2.4
**Cutaneous nodules**	48.9	48.2	2.9	46.1	53.9	0	27.6	69	3.4
**Renal disease**	16.2	79.1	4.7	2.8	97.2	0	4.1	90	5.9
**Ocular lesions**	25.8	71.1	3	5.5	89.5	5	18.2	0	81.8
**Epistaxis**	30.1	68.2	1.6	7.5	91.9	0.5	40	6.7	53.3
**Arthropathies**	42.5	56.3	1.3	16	83.4	0.6	59.2	40.3	0.5
**Fever**	38.6	58.6	2.9	41.4	58.6	0	35.9	63.2	1
**Diarrhoea**	58.1	40.9	0.9	33.5	66.5	0	38	60.2	1.9
	**Slovenia n = 49**			**Spain n = 483**			**Portugal n = 141**		
**Weight loss**	0	81.8	18.2	3.1	70.8	26.1	1.4	66.2	26.6
**Alopecia**	0	100	0	1.6	85.9	12.5	1.4	80.6	10.1
**Lymphadenomegaly**	0	100	0	13.0	61.3	25.7	2.9	69.8	18.0
**Lethargy**	0	81.8	18.2	3	90.4	6.7	5.8	77.0	3.6
**Pale mucosa**	0	90	10	4.3	87.7	8	6.5	76.3	4.3
**Exfoliative dermatitis**	9.1	90.9	0	2.8	82.8	14.4	3.6	77.0	6.5
**Onychogryphosis**	0	100	0	5.7	85.8	8.5	3.6	81.3	5.8
**Skin ulcers**	0	100	0	5.5	88	6.4	7.2	79.9	2.2
**Pyodermatitis**	0	90.9	9.1	7.3	86.7	6.1	8.6	78.4	0.7
**Footpad lesions**	0	100	0	13.2	84.2	2.6	10.8	76.3	1.4
**Cutaneous depigmentation**	0	100	0	21.9	75.4	2.7	2.9	53.2	3.6
**Cutaneous nodules**	0	100	0	23.8	73.7	2.5	20.1	48.9	15.8
**Renal disease**	0	100	0	7	89.1	4	3.6	85.6	1.4
**Ocular lesions**	0	90	10.0	7.9	87.1	4.9	10.1	77.7	1.4
**Epistaxis**	11.1	88.9	0	8.2	73	18.8	8.6	79.1	0.0
**Arthropathies**	0	100	0	13.2	84.9	1.9	21.6	64.7	0.7
**Fever**	0	90.9	9.1	38.7	60.5	0.7	32.4	54.0	0.0
**Diarrhoea**	0	90.9	9.1	30.2	69.3	0.5	33.1	51.8	0.0

An important aspect of this section was to describe the presence of diseases associated with CanL. As associated diseases, the clinicians reported vector-borne, parasitic or infectious diseases, endocrinopathies, neoplasias, autoimmune and metabolic diseases. The most frequent coinfections mentioned were canine ehrlichiosis (51%), followed by other vector-borne diseases: dirofilariosis (12%) and babesiosis (6%); parasitic diseases: demodicosis (8%) and sarcoptic mange (6%), and infectious diseases: dermatophytosis (4%). It should be noted that the Greek participants reported the highest incidences of coinfections with canine ehrlichiosis (86%) and dirofilariosis (22%), and the French clinicians described CanL mostly associated with demodicosis (14.8%), sarcoptic mange (14.8%) and dermatophytosis (8.7%).

### Diagnostic methods

When leishmaniosis was suspected, specific tests were employed to confirm a diagnosis of CanL. The diagnostic methods asked about were classified into: cytology and microscopic examination (skin lesions, lymph nodes or bone marrow), serological tests (IFAT, ELISA and rapid tests) or other techniques (PCR, histopathology, immunohistochemistry and protein electrophoresis) (Table 4).

**Table 4:** Diagnostic tools used by veterinarians to confirm a suspected case of CanL

	**France n = 994**			**Greece n = 201**			**Italy n = 231**		
**Technique %**	**Never**	**Occasional to frequent**	**Always**	**Never**	**Occasional to frequent**	**Always**	**Never**	**Occasional to frequent**	**Always**
**Cytology**									
**Lymph nodes**	34.6	52.8	12.6	21.4	58.6	20	46.7	48.0	5.3
**Bone Marrow**	65.5	30.6	3.9	69.6	29.4	1.1	59.9	33.6	6.6
**Skin lesions**	65	28.9	6.1	56.6	40.4	3	28.8	56.5	14.7
**Serology**									
**IFAT**	22.7	28.7	48.6	16	45	39	4.3	48.9	46.8
**Rapid tests**	42	33.9	24	4.6	37.1	58.3	30.9	38.2	30.9
**ELISA**	13.5	34.7	51.9	14.3	58.9	26.8	25.2	37.7	37.1
**Other techniques**									
**PCR**	55.7	36.6	7.7	52.1	46.4	1.4	20.9	66.9	12.2
**Protein electrophoresis**	42.3	41	16.8	76.6	20.4	2.1	10.6	39.8	49.7
**Histopathology**	51.1	45.8	3.1	72.3	27.7	0	64.9	35.1	0
**Immunohistochemistry**	94.3	4.7	1	94.5	5.5	0	83	13.8	3.2
	**Slovenia n = 49**			**Spain n = 483**			**Portugal n = 141**		
**Cytology**									
**Lymph nodes**	2	4	0	14.3	80.2	5.5	19.4	72.1	8.6
**Bone Marrow**	2	2	0	18.1	71.4	10.5	36.2	53.2	10.6
**Skin lesions**	6.1	2	0	34.2	61.8	3.9	82.9	14.6	2.4
**Serology**									
**IFAT**	0	4	0	6.3	37.9	55.9	9.4	36.7	25.9
**Rapid test**	0	2	2	11.5	54.0	34.5	20	43.5	36.5
**ELISA**	0	2	6.1	42.3	47.3	10.4	43.9	43.9	12.2
**Other techniques**									
**PCR**	0	2	2	8.3	86.6	5.1	57.8	37.6	4.7
**Protein electrophoresis**	0	2	0	nd	Nd	nd	96.5	1.8	1.8
**Histopathology**	0	4.1	0	35.3	63.9	0.8	91.5	6.8	1.7
**Immunohistochemistry**	0	2	0	15.0	40.4	44.6	33.8	48.8	17.5

nd: not determined.

**Figure 2:**
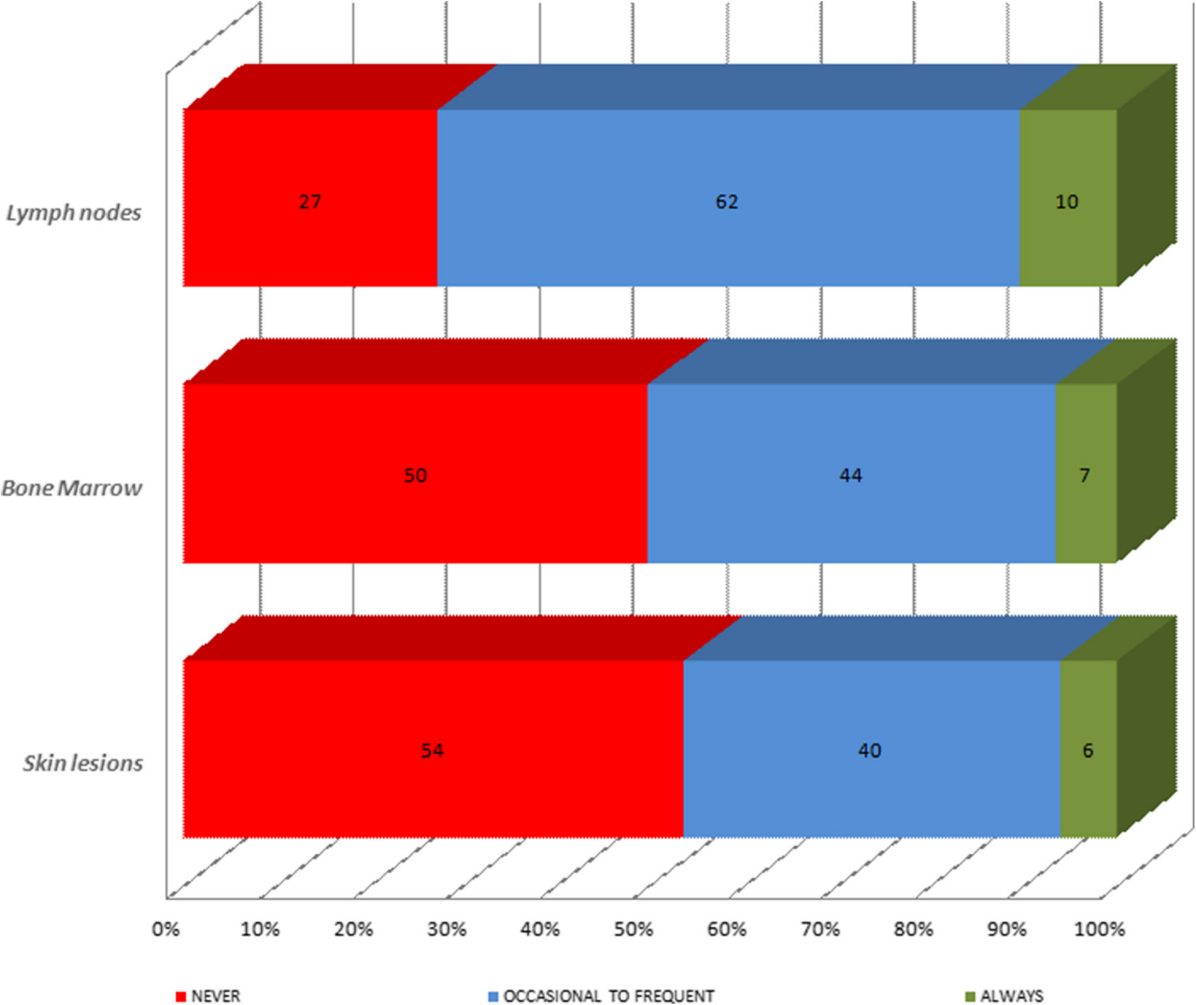
Cytology options used to diagnose CanL reported by veterinarians from Italy, Portugal, Spain, Greece and France.

**Figure 3:**
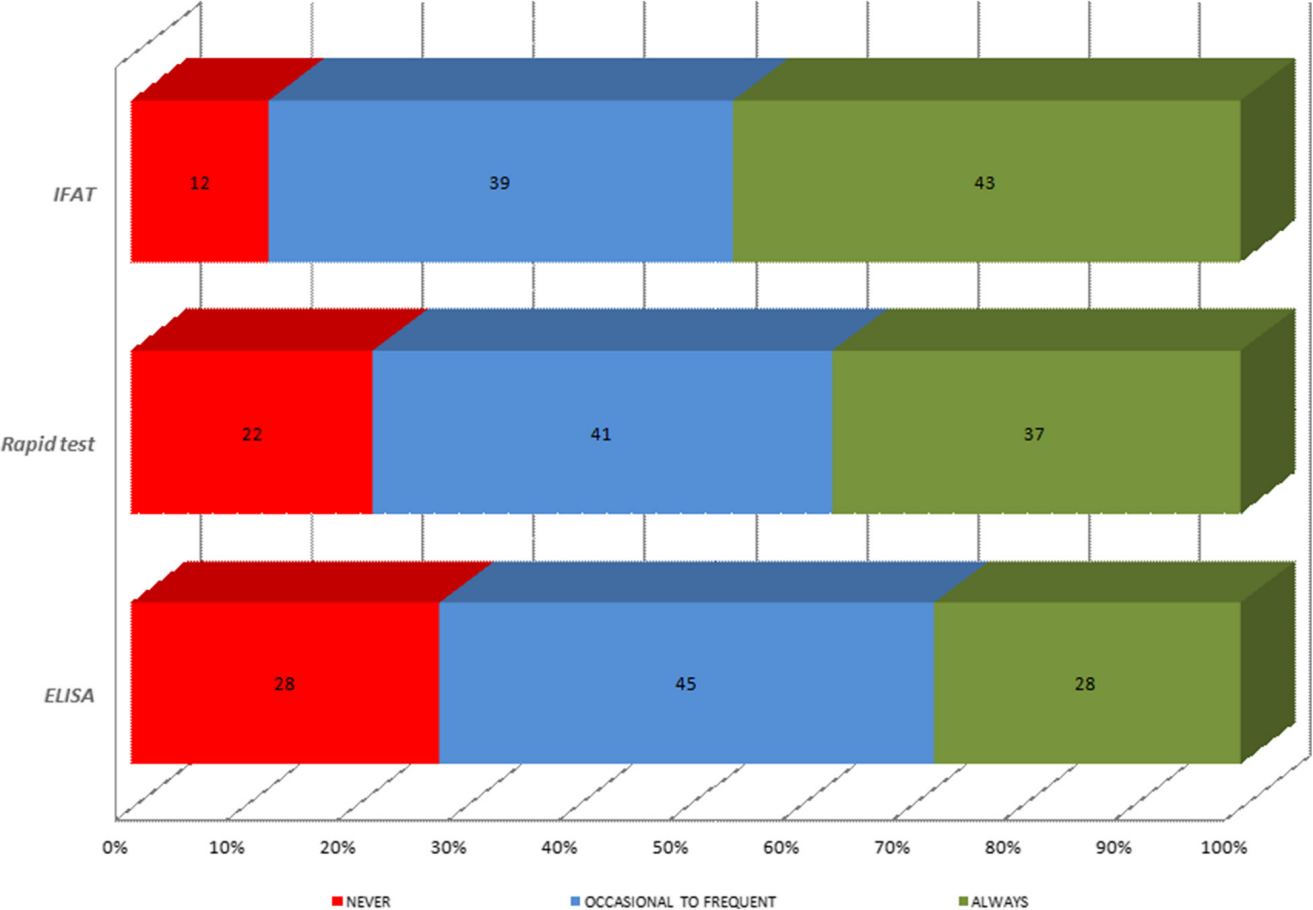
Serology techniques used to diagnose CanL reported by veterinarians from Italy, Portugal, Spain, Greece and France.

**Figure 4:**
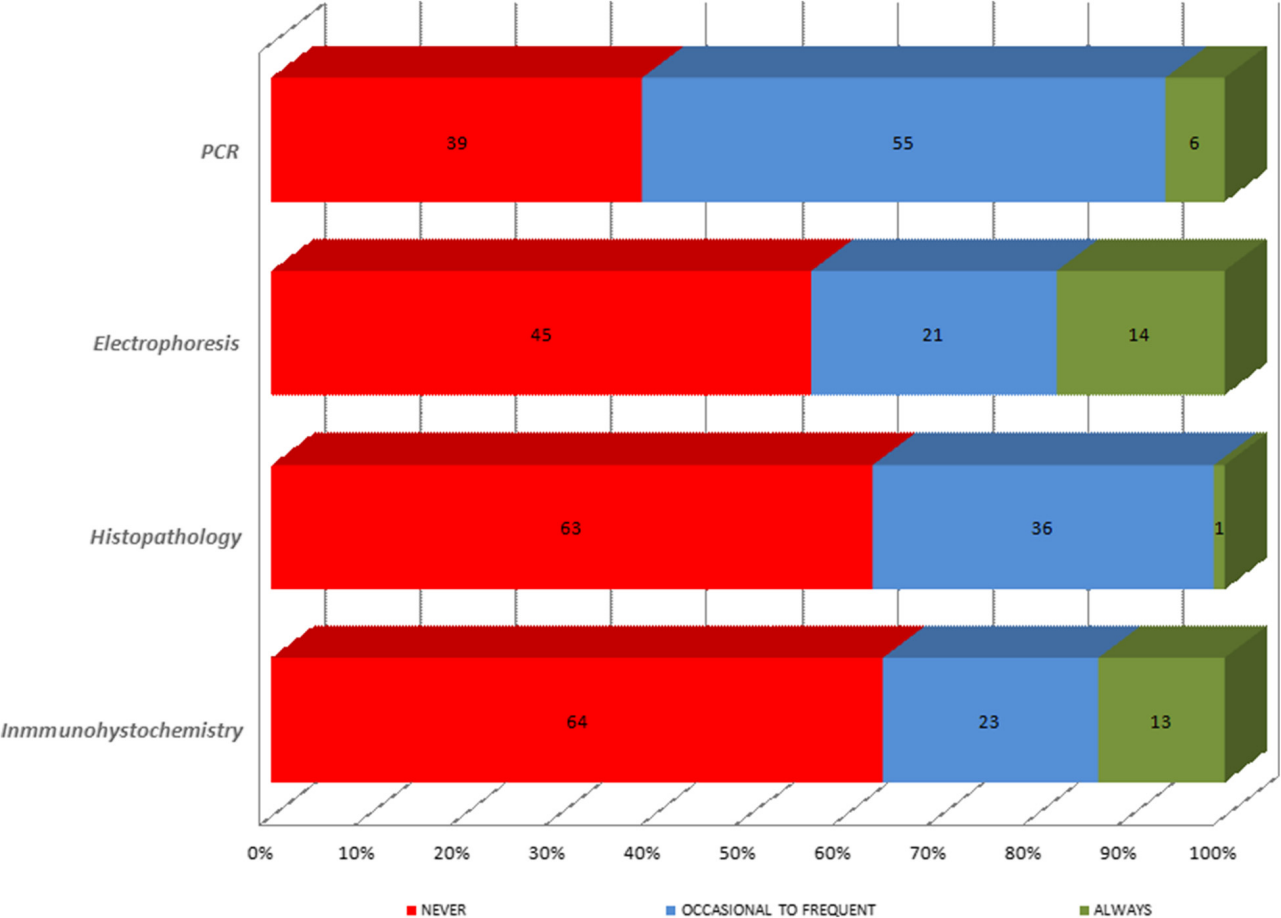
Other techniques used to diagnose CanL reported by veterinarians from Italy, Portugal, Spain, Greece and France.

Veterinarians reported microscopy examinations of lymph node aspirates as their first choice etiological diagnostic method (Figure 2). Cytology samples obtained from bone marrow and skin lesions showed good acceptance but were less used. Veterinarians who would never use an invasive technique for a bone marrow cytology were predominant in France (65.5%), Greece (69.6%) and Italy (59.9%) and those who never examined skin samples were predominant in Portugal (82.9%) and France (65%). As illustrated in Figure 2, an in-house etiological diagnosis consisting of microscopy observation of potentially infected tissue was not of routine use. Accordingly, a minority of veterinarians reported they always used a cytological method: lymph nodes (10%), bone marrow (7%) and skin lesions (6%). As complementary tests, microscopy observation of lymph node aspirates was the most used occasionally to frequently (62%). The quantitative serological method most used was IFAT (82.7%), followed by qualitative in-house rapid tests (78.2%) and ELISA (72.2%). For routine use, IFAT and qualitative rapid tests were described as always employed by 43% and 37% of veterinarians respectively (Figure 3). Qualitative rapid tests were most used for a routine diagnosis in Greece (58.3%), Portugal (63.5%), Spain (34.5%) and Italy (30.9%). Lastly, among the “Other techniques”, PCR was most reported (61%). Protein electrophoresis (14%) and immunohistochemistry (13%) were used more in routine practice than PCR (6%) or histopathology (1%) (Figure 4).

### Treatment and follow-up of CanL

**Figure 5:**
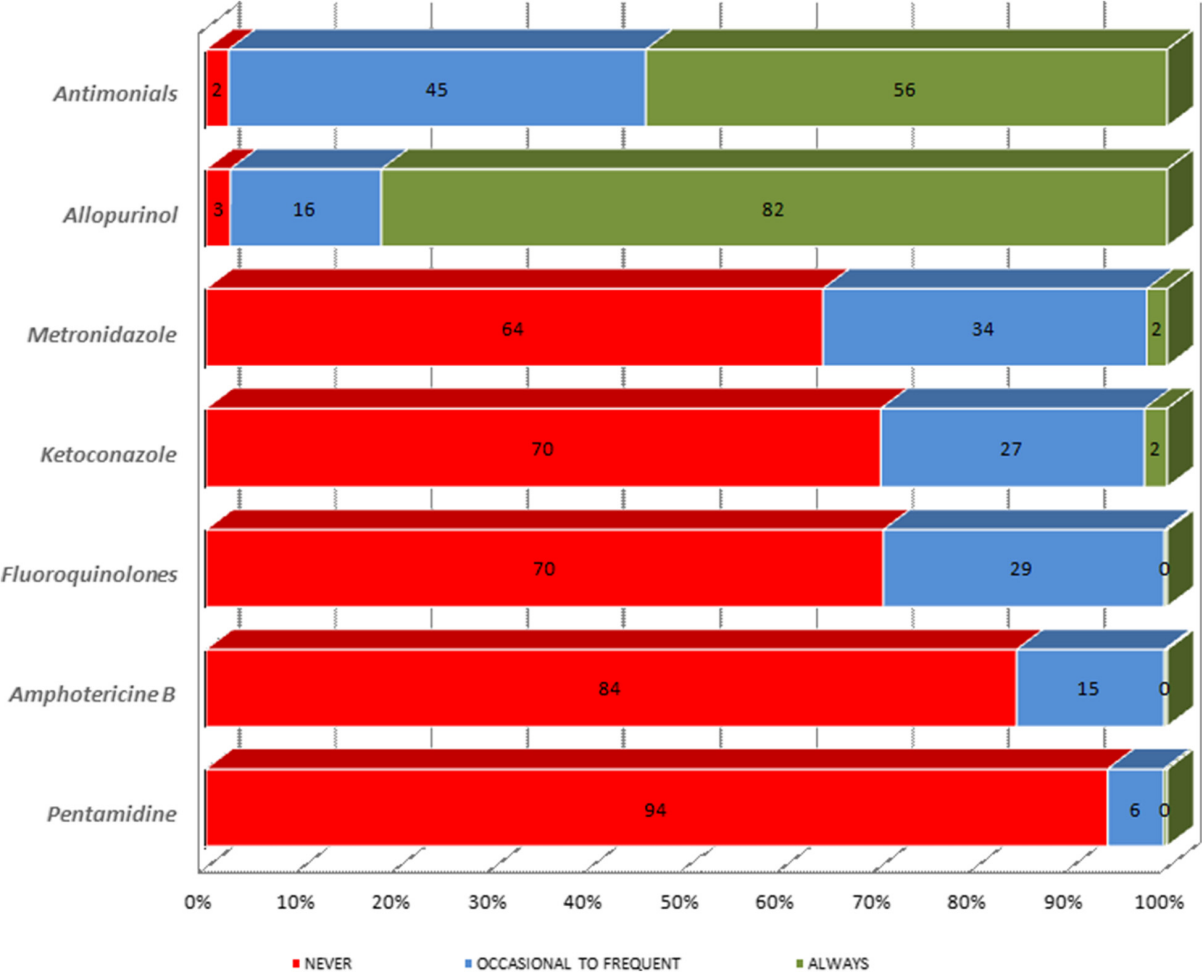
Active ingredients used by veterinarians to treat confirmed cases of CanL in Italy, Portugal, Spain, Greece and France.

For this section of the questionnaire, we report and summarize the therapeutic regimens and agents selected by the veterinarians. The agents proposed were: antimonials, allopurinol, amphotericin B, pentamidine, ketoconazole, metronidazole and fluoroquinolones. Those most used were antimonials and allopurinol, the latter being the most frequently used (Figure 5).

The regimens included in the questionnaire were: antimonials plus allopurinol, allopurinol alone, antimonials alone and amphotericin B (Table 5). Most vets in endemic countries used mostly the combination antimonials and allopurinol to treat CanL, except for Greece and Portugal where allopurinol alone was the first treatment option. Allopurinol alone was chosen as first line treatment by the Portuguese, Greek and Slovenian clinicians. Despite their lower efficacy, antimonials alone were the treatment of choice for 13.7% to 17.5% depending on the country, and were used as second line treatment by the Spanish and Italian vets. The use of amphotericin B was not an option for the European practitioners (0.4-3.1%).

**Table 5:** Therapy protocols used by veterinarians to treat confirmed CanL cases

**Protocol %**	**France**	**Greece**	**Italy**	**Slovenia**	**Spain**	**Portugal**
**Antimonials + allopurinol**	80.8	24.9	65.4	2	58.6	68
**Allopurinol only**	23.3	31.3	12.1	6.1	8.5	84
**Antimonials only**	17.5	16.9	13.9	0	13.7	nd
**Amphotericin B only**	0	1.5	0.4	0	3.1	nd

nd: not determined.

Survival times of treated animals were longer in Spain, Portugal, Italy and France. In Greece, the expected time of survival was shorter. The data from Slovenia was insufficient to draw any conclusions on this point (Table 6).

**Table 6:** Survival times of dogs after treatment

**Survival rate %**	**France**	**Greece**	**Italy**	**Slovenia**	**Spain**	**Portugal**
**< 3 months**	8.6	0.19	9.6	2	0	0
**3-6 months**	4.7	38.9	11	2	1	1
**6 m – 1 year**	7.2	48.1	12.5	2	4	3
**1-2 years**	13.5	25.8	21.5	2	8	10
**2-5 years**	35.7	8.2	42.8	2	48	58
**> 5 years**	30.3	7.1	45.1	0	39	38

### Preventive measures and public health implications

In this section of the questionnaire, the veterinarians were asked about their recommendations to dog owners about the use of topical insecticides if the dogs were living in or moving to an endemic area. The veterinarians were also asked about other prophylactic measures they recommended for dogs living in endemic areas: keeping indoors at night, the use of fine-mesh nets and/or environmental insecticides.

The replies given to the questions in the prophylaxis section are provided in Table 7. Most vets recommended the use of insecticides and repellents to prevent the spread of CanL for a dog living in an endemic area (91.6%) or moving to an endemic area (83.7%). The most recommended preventive measure by vets was that dogs should be kept indoors at night (45.4%) followed closely by the use of fine-mesh nets (31.7%).

**Table 7:** Preventive measures recommended by veterinarians in endemic and non-endemic areas of CanL and public health implications

**Preventive measures**														
**Question answered**	**France %**		**Greece %**		**Italy %**		**Slovenia %**		**Spain %**		**Portugal %**		**Total**	
	Yes	No	Yes	No	Yes	No	Yes	No	Yes	No	Yes	No	Yes	No
**1**	94.8	5.2	96.9	3.1	98.6	1.4	73.5	2	90.1	0	96	4	91.6	2.6
**2**	93.6	6.4	95.7	4.3	75.2	24.8	55.1	2	87.5	0.8	95	5	83.7	7.2
**3**														
**3.1**	64.1	35.9	57.4	42.6	50.7	49.3	Nd	nd	nd	nd	100	0	45.4	21.3
**3.2**	nd	nd	48.9	51.1	41.3	58.7	Nd	nd	nd	nd	100	0	31.7	18.3
**3.3**	4.1	95.9	13.2	86.8	8	92	Nd	nd	nd	nd	nd	nd	4.2	45.8
**Vaccination**														
**4**	98.7	1.3	90.8	0.0	100	0	Nd	nd	100	0	95	5	96.9	1.3
**5**	94	6	93	0.0	100	0	Nd	nd	99	1	95	5	96.2	2.4
**6**	92.9	7.1	91.2	8.8	72.6	27.4	Nd	nd	92.7	0.3	100	0	89.9	8.7
**Public health implications**														
**7**	73.6	26.4	98.5	1.5	93.5	6.5	40.7	8.2	94.6	5.4	63	37	77.3	14.2
**8**	88.6	11.4	100	0	96.5	3.5	46.9	6.1	97.8	2.2	40	60	78.3	13.9
**9**	4	96	7.6	92.4	13.7	86.3	0	61.2	14.3	85.7	0	100	6.6	86.9

**Questions: (1)** Do you recommend the use of insecticides/repellents for dogs living in an endemic area?**, (2)** Do you recommend the use of insecticides/repellents for dogs moving to a non endemic area?**, (3)** Do you recommend any other prophylactic measures for dogs in an endemic area?**, (3.1)** Keeping dogs indoors at night**, (3.2)** Fine-mesh nets**, (3.3)** Environmental pesticides**, (4)** Would you use an effective and safe vaccine for dogs living in an endemic area?**, (5)** Would you use an effective and safe vaccine in a dog moving to an endemic area?**, (6)** Would you use an effective and safe vaccine in combination with other prophylactic measures?, **(7)** Are dog owners aware of the public health implications of CanL?, **(8)** Do you inform owners about the public health implications of CanL?, **(9)** Are you aware of any cases of simultaneous dog-owner infection?; **nd**: not determined.

Most vets questioned stated they would use an effective and safe vaccine, if available, in dogs living in endemic areas (96.9%) and dogs travelling to an endemic area (96.2%), even combined with other prophylactic measures (89.9%) (Table 7).

Most clinics informed their clientele about the public health implications of CanL (77.3%). The southern European inhabitants were sufficiently informed about this topic (78.3%). In the case of Portugal, these figures were only 63% and 40% respectively. Very few vets were aware of the possibility of simultaneous dog-owner infection (6.6%).

## Discussion

The questionnaire used in our survey was designed to obtain data about the current situation of CanL in SW European countries. This is the first multinational approach to the collection of field data and comparative information on CanL. Data collected from similar questionnaires have provided good background information on the distribution and management of CanL [43-46].

The size of a veterinary clinic and number of employees reflects the quality of service provided [47]. Hence, the presence of many small clinics and practices in Italy, Greece, Portugal and Slovenia with limited resources indicates a more basic veterinary service provided by professional veterinarians. In contrast, countries like France and Spain have a large number of well-equipped veterinary clinics and centres, determining a quantitative improvement in the degree of specialization. The presence of larger veterinary clinics is an indicator of specialization and of a wider knowledge of the corresponding staff. France emerged as the country with the larger proportion of veterinary hospitals involved in completing the questionnaire.

The issues discussed hereafter relate to the information provided by all the questionnaires except those completed by the Slovenian vets. The reason for this is the scarce knowledge of CanL in this country and the limited number of reported cases, which suggests a need for further studies in non-endemic areas to evaluate the potential risk of developing this disease [48].

According to the average numbers of CanL cases per year reported for each country, the well known endemic areas Greece, Italy, Portugal, Spain and southern France emerged as leishmaniosis hot spots [10].

The broad spectrum of clinical signs of CanL hinder and lengthen the process of its clinical diagnosis [18]. In endemic areas, a single sign compatible with CanL should justify confirmation of the infection [17]. In this survey, the most common clinical signs reported were: weight loss, alopecia, lymphadenomegaly, lethargy, pale mucosa, renal disease and a large number of skin lesions. These are all strong clinical indicators of CanL as widely reported in the scientific literature [8,19,49] and reported by other authors [43-46]. The clinical signs that we called “early detectable signs” were the most important indicators of the presence of the disease in terms of their high frequency and diagnostic value. Weight loss, alopecia and lymphadenomegaly have been frequently observed in an objective and thorough physical examination of the dogs [50-53]. Nevertheless, the factors lethargy or pale mucosa, which is caused by a non-regenerative anaemia, are not so definitive for diagnosing the disease since they are subjective and transient, respectively [54,55]. Cutaneous lesions were often observed but are not specific to CanL and may be caused by many other diseases such as autoimmune diseases. Clinical signs of high prognostic value are those associated with immunocomplex deposition at the renal glomeruli [56], joints [57], eyes [58-60] or blood vessels [61]. These signs are a severe manifestation of disease progression, renal disease being the more frequently reported severe form [62] and the first cause of death in dogs with CanL. Fever and diarrhoea are unspecific, uncommon and were described as incompatible with CanL by the responding practitioners. Fever may indicate the presence of another infectious disease (mainly vector-borne), autoimmune disease or a toxic event. Diarrhoea is also caused by gastrointestinal parasites, inflammatory disease, food allergy or a gastrointestinal disorder. Diarrhoea is an infrequently observed clinical sign of CanL and has only been reported in cases of ulcerative colitis [63,64].

The management of CanL should include a differential diagnosis to rule out other vector-borne diseases (eg, ehrlichiosis, babesiosis) or diseases producing skin lesions (eg, autoimmune diseases) [18]. Veterinarians should also keep in mind that many dogs with CanL could have concomitant disorders linked to their immunocompromised state or due to another vector-borne disease [65,66]. In the present survey, the disease most frequently associated with CanL was canine ehrlichiosis, and this was followed by many other parasites (eg, dirofilariosis, babesiosis, demodicosis) or infectious (eg, bacterial dermatoses, dermatophytosis) diseases [67]. The presence of other canine vector-borne diseases is directly related to the vector distribution in southern European countries. It should nevertheless be considered that some vector borne diseases like *Bartonella* spp. or *Anaplasma* spp. infection are still underdiagnosed and that affected animals could be coinfected with *Leishmania infantum*[68]. Greece provided the higher number of coinfections with canine ehrlichiosis and dirofilariosis. This may be attributed to the high endemicity of these diseases due to appropriate climate conditions in this country. In France, cases of CanL were more associated with demodicosis, sarcoptic mange and dermatophytosis, probably due to the traditional expertise of French veterinarians in dermatology. These findings could thus depend on the geographical location of the country and the interest of the different veterinarians questioned.

When CanL was suspected, specific tests were employed to confirm its diagnosis. Observation of stained lymph node aspirates by microscopy was employed by veterinarians more frequently than observation of samples obtained from bone marrow aspirates or skin cytologies. As routine tests, practitioners rarely undertook the microscopic detection of amastigotes in targeted tissues since cytology is usually more frequently performed by a consulting specialist. Although the direct observation of the parasite is a conclusive diagnosis, microscopy shows a low sensitivity and its specificity depends on parasite load, observer skill and the time dedicated to examining smears [6,69]. Not surprisingly, invasive techniques to obtain bone marrow aspirates was infrequently reported because of the skill required and the need for sedation, which is time consuming and costly. In contrast, the scarce use of skin cytology among the vets questioned is surprising.

Serological tests were widely used by the clinicians. The diagnostic method most used was IFAT. In addition, IFAT was the most widely used routine technique. A reliable diagnosis of CanL requires quantitative serological methods guided by an exhaustive evaluation of clinical signs and clinicopathological abnormalities [18,23]. Quantitative serology is appropriate for diagnosis in clinical practice and for treatment follow-up [18,23,29]. However, it has to be performed in specialized laboratories. A high percentage of the vets (37%) reported the routine use of qualitative rapid tests. Rapid tests are not able to identify dogs with low antibody levels so a positive result by a qualitative test is only a first step and must be followed by a quantitative serological method. This is recommended in the Leishvet guidelines [18,19] for precise antibody titration, a good prognosis and therapy monitoring. In the section “Other techniques”, PCR was the technique most widely accepted. PCR is an expensive technique only performed in specialized laboratories. It is not used alone for diagnostic purposes; a positive PCR diagnosis indicates *L. infantum* infection but not necessarily clinical disease in the dog [18]. Real-time PCR is an advanced technique that can quantify *Leishmania* loads in infected dog tissues. However, although it is useful for diagnosis and the follow-up of treated dogs [29,70,71], its results are difficult to interpret due to their inconsistency such that very sick dogs may show low parasite loads and vice-versa. Under this same section, the reported use of routine diagnostic tests such as protein electrophoresis and immunohistochemistry should help improve the diagnosis of CanL [21,72].

The antileishmania agents most used by the vets surveyed were antimonials and allopurinol, the latter being the most frequently employed. The first treatment option was meglumine antimoniate, which is usually associated with allopurinol. These drugs have a synergistic activity when combined [26,27,73]. Despite the fact that some vets used antimonials alone, this is currently not recommended owing to a reported increase in clinical disease recurrence [74]. Amphotericin B was fortunately not an option for practitioners; it is the first line option to treat human leishmaniosis and its cumbersome endovenous administration route makes it unsuitable for veterinary use. Moreover, this molecule is poorly tolerated by dogs. It should be noted that when this survey was conducted, miltefosine, was still not licensed in Europe and thus none of the clinicians reported its use.

The dosing regimen used to treat CanL differed among the countries. In France, antimonials were administered at a dose of 100–200 mg/kg sc (subcutaneously) for 20–30 days combined with allopurinol at a dose of 10–30 mg/kg po (orally) for several months, or for 7–10 days per month or even for life. In Spain, antimonials were administered as 50–100 mg/kg bid (twice a day) (89%), combined with allopurinol at a dose of 20 mg/kg bid for 3–6 months (39%) or 12 months (10%) and even for life (10%). In Greece, the antimonial dose used was 50–300 mg/kg sc for 20–80 days in combination with allopurinol at a dose of 7–40 mg/kg (15 mg/kg of average) for 6 months. In Portugal, dosing regimes varied from 10–20 mg/kg/sid (once daily) for allopurinol alone or in association with meglumine antiamoniate at 100 mg/kg/sid for 1 month. Allopurinol was administered during a minimum of 3 months to lifelong treatment and was considered a safe drug. Meglumine antiamoniate side-effects were occasionally noticed (36%) and included vomiting, diarrhoea, anorexia, lethargy and pain at the injection site. All other treatments (amphotericin B, lomidine, ketoconazole, metronidazole, antibiotics) were rarely prescribed. In Italy, antimonials were administered at a dose of 100 mg/kg bid (71%) sc (63%) combined with allopurinol at a dose of 10 mg/kg bid for 3–6 months (84%), or for 12 months (7.5%) and even for life (11%). In Slovenia, the majority of practitioners used allopurinol alone, while antimonials were periodically used only in one Slovenian practice. The rest of the practitioners rarely considered the use of antimonials because of their high cost and administration route. In contrast, allopurinol is cheap and used orally which is why many Slovenian vets use it on its own. It should be mentioned that dosing regimens for efficient agents against CanL vary widely and there are usually over 15 protocols in a single country for a given drug (eg, Spain antimonials plus allopurinol = 17 and allopurinol = 13; Italy antimonials plus allopurinol = 26 and allopurinol = 11). This situation suggests a need to standardize both the choice of the most appropriate drug and its dosing regimen. Surprisingly, the doses of agents used to treat CanL reported by a few veterinarians were three times the standard recommended dose. In an effort to standardize the wide variety of agents and protocols used in Europe, the LeishVet group has established a set of guidelines detailing the most effective doses and drugs against CanL according to evidence based medicine [18,19].

The survival rates provided by the vets must be interpreted as self-perceived rates. Notwithstanding, survival times are getting longer [75] due to improved clinical management and to new preventive tools such as topical repellents, vaccines or immunomodulators. Expected survival is 2–5 years after primodiagnosis, except for chronic patients with signs of associated immunocomplex deposition [76].

Finally, the veterinarians properly informed dog owners about the preventive measures available and about the zoonotic implications of CanL. Almost all veterinarians actively recommended the topical use of insecticides/repellents as reported in other European countries [43-46,77]. Avoiding sand fly bites is useful in preventing *L. infantum* infection in dogs and could reduce the incidence of the disease [34,78].

The veterinarians were also asked about other prophylactic measures they would recommend for dogs in endemic areas: keeping dogs indoors at night or the use of fine-mesh nets and/or environmental insecticides. The most recommended preventive measure was that dogs should be kept indoors at night. Spending the night outdoors has been described as a risk factor for infection by *L. infantum* because dogs are exposed to sand flies for a longer time period [79,80]. The use of fine-mesh nets is an effective strategy against sand fly exposure [81]. Most vets recommended the use of an effective and safe vaccine for dogs in endemic areas (97.4%), moving to an endemic area (96.5%) or even combined with other prophylactic measures (94.2%). Used in combination with topical repellents, vaccination is a promising tool for controlling CanL [36]. Veterinarians should always insist on informing dog owners of the health implications of CanL in their country. It should be mentioned that health education and knowledge of the disease in Portugal was the poorest among the countries examined where CanL is endemic.

In summary, this paper provides a global picture of the current situation of the clinical management of CanL in six SW European countries based on data reported by local veterinarians from each region.

## Conclusions

This survey collates data on the current situation regarding the clinical management of CanL in SW European countries. The highly variable data provided by veterinarians from each country suggest a need to follow standardized guidelines indicating the best diagnostic and treatment options for this disease.

## Competing interests

The authors declare no competing interests.

## Authors’ contributions

PB designed and distributed the prototype questionnaire to academics from European Veterinary Schools of the six countries included in the study, reviewed the first version of the manuscript and finalized the manuscript. GM compiled the data from the six countries involved, constructed the tables, drafted and reviewed the first version of the manuscript and finalized the manuscript. RG constructed the figures, drafted the first version of the manuscript and finalized the manuscript. GM distributed the questionnaire to Spanish veterinarians, translated it into Spanish and prepared the country’s results report. MS and AK distributed the questionnaire to Greek veterinarians, translated it into Greek and prepared the country’s results report. AO and IPF distributed the questionnaire to Portuguese veterinarians, translated it into Portuguese and prepared the country’s results report. GO and VM distributed the questionnaire to Italian veterinarians, translated it into Italian and prepared the country’s results report. TK distributed the questionnaire to Slovenian veterinarians, translated it into Slovenian and prepared the country’s results report. All authors reviewed the manuscript. All authors read and approved the final manuscript.
